# Predictive factors for remission in post-stroke depression: A Markov model cohort study

**DOI:** 10.1016/j.isci.2026.116438

**Published:** 2026-06-17

**Authors:** Wenwen Liang, Yifan Fang, Tianyi Li, Qing Du, Yingjie Liu, Siyi Chen, Chensheng Pan, Guo Li, Zhou Zhu

**Affiliations:** 1 Department of Neurology, Tongji Hospital, Tongji Medical College, Huazhong University of Science and Technology, Wuhan, Hubei 430030, China

**Keywords:** health sciences, psychology

## Abstract

Post-stroke depression (PSD) follows a dynamic remission-relapse course, yet predictors of state transitions remain unclear. Here, we applied a continuous-time multi-state Markov model to 536 ischemic stroke patients with 1,441 longitudinal assessments. We quantified transition intensities between non-PSD, mild-PSD, and severe-PSD states and identified baseline predictors of deterioration and recovery. The transition intensity from mild to non-PSD was 5.5-fold higher than progression to severe-PSD. Education ≥10 years reduced deterioration risk (HR = 0.63), while functional independence (Barthel index >60) and social support predicted remission. Severe-PSD exhibited an apparent short sojourn time (estimated mean 2.7 months) and frequent observed transitions toward milder states; however, in the absence of mortality data, this estimate may be biased by unobserved competing events and should be interpreted as hypothesis-generating rather than definitive. These findings enable early risk stratification and targeted intervention for PSD management.

## Highlights

• Post-stroke depression states exhibit bidirectional dynamic transitions
• Recovery from mild-PSD occurred more readily than progression to severe states
• Education and social support were associated with reduced depression risk
• Severe-PSD showed short sojourn time and high observed exit rates

## Graphical abstract

**Figure:**
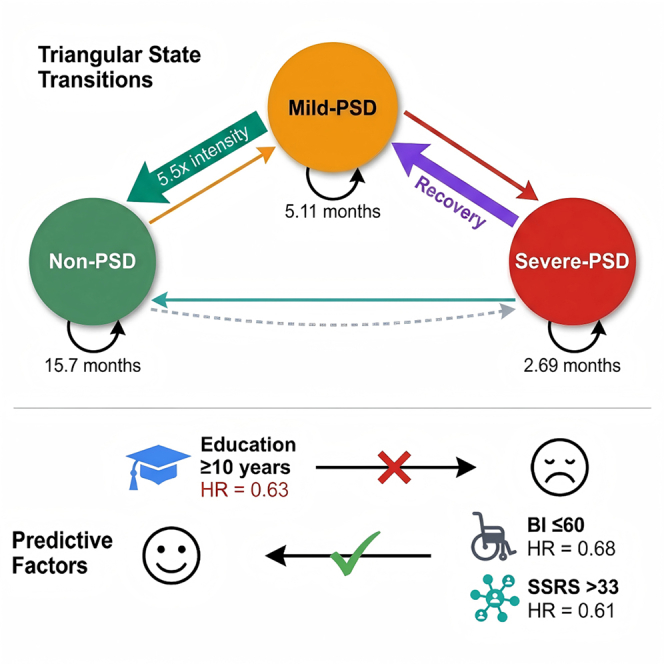

## Introduction

Post-stroke depression (PSD) is considered the most common and most harmful psychological complication among stroke survivors.^1^ There is sufficient evidence indicating that PSD is closely related to impaired functional ability, reduced quality of life, and increased mortality.^2^ PSD is a process that follows a long-term natural course of remission-relapse, with about one-third of survivors experiencing remission without recurrence. Therefore, understanding the longitudinal development pattern of PSD is very important.^3^ However, existing research has critical limitations: one is that it focuses on different cross-sectional time points and methods to describe the prevalence of PSD and its related factor analysis, without considering its dynamic changes over time^4^; Second, few studies use group-based trajectory models to determine the natural trajectories of PSD, which cannot accurately predict the reversal and progression of PSD relatively quantitatively^5^; Third, few studies have explored the factors influencing positive outcomes of depression (e.g., from presence to absence, or from severe to mild depression), although this is crucial for designing interventions to improve patients’ long-term prognosis.

The pathogenesis of PSD is multifactorial, typically including factors such as age, gender, stroke severity, education level, marital status, and social support.^6^^,^^7^ Although previous studies have found strong correlations between these factors and PSD, their role in driving specific PSD transitions remains unclear. Additionally, most existing longitudinal studies were designed specifically to investigate the factors influencing the progression of PSD (from none to presence), with limited literature on the reversal of recovery.^8^ Understanding the reversible potential of individual transitions under specific disease states for these covariates have considerable public health significance in preventing adverse health outcomes related to PSD.

Multi-state Markov model (MSM) can effectively overcome the limitations of traditional regression analysis and stratified models in capturing the dynamic nature and threshold effects of specific diseases.^9^ This model not only reflects the transition strength between any two states but can also predict the result state during subsequent visits by using the covariate values from the previous visit, effectively determining the predictive variables for state transitions. For example, the MSM model can describe in detail how individuals transition to various stages of the disease, incorporating individual heterogeneity. The MSM model has been successfully applied to describe state transitions of various diseases, including sarcopenia,^10^ body weight status,^11^ depressive symptoms,^12^ and mild cognitive impairment transitions.^13^ However, despite the significant achievements of MSM in multiple disease areas, the inter-state transitions and influencing factors of PSD, as a dynamically developing disease, remain unclear.

Based on this, this study uses the MSM model to estimate the progression and reversal process of PSD and provides information on the instantaneous transition intensity and probability between PSD states, the impact of covariates on transitions, and the estimated average residence time, with the aim of providing a scientific basis for the prevention and management of PSD and assisting in the development of more accurate intervention strategies.

## Results

### Baseline characteristics of cohort samples

A total of 536 participants were included, with 78.9% being male and an average age of 59.13 ± 11.07 years. The baseline PSD status of the participants was as follows: 301 people (56.2%) were non-PSD, 180 (33.6%) had mild-PSD, and 55 (10.3%) had severe-PSD. Table 1 summarizes the baseline demographic and clinical characteristics of these individuals. Among them, compared to those without depression and with mild depression, the severe-PSD group had a higher proportion of females, higher National Institutes of Health Stroke Scale (NIHSS) and modified rankin scale (mRS) scores, and lower Barthel index (BI) and social support rating scale (SSRS) scores.

**Table 1:** Baseline characteristics of the participants

Variables	Total (*n* = 536)	Non-PSD (*n* = 301)	Mild-PSD (*n* = 180)	Severe-PSD (*n* = 55)	P
Sociodemographic parameters					
Sex = Male (%)	423 (78.9)	244 (81.1)	145 (80.6)	34 (61.8)	0.005
Age (mean (SD))	59.13 (11.07)	59.77 (11.00)	58.39 (11.37)	58.07 (10.30)	0.313
BMI (mean (SD))	24.79 (3.90)	24.93 (3.99)	24.93 (3.83)	23.61 (3.42)	0.058
Education (%)≥10 years	228 (42.5)	137 (45.5)	67 (37.2)	24 (43.6)	0.202
Married (%)	526 (98.1)	295 (98.0)	177 (98.3)	54 (98.2)	0.967
Vascular risk factors					
Smoking = Yes (%)	244 (45.5)	148 (49.2)	76 (42.2)	20 (36.4)	0.119
Drinking = Yes (%)	227 (42.4)	122 (40.5)	82 (45.6)	23 (41.8)	0.557
Diabetes mellitus = Yes (%)	152 (28.4)	93 (30.9)	47 (26.1)	12 (21.8)	0.278
Hypertension = Yes (%)	315 (58.8)	184 (61.1)	105 (58.3)	26 (47.3)	0.157
Dyslipidemia = Yes (%)	132 (24.6)	72 (23.9)	44 (24.4)	16 (29.1)	0.714
CHD = Yes (%)	50 (9.3)	24 (8.0)	18 (10.0)	8 (14.5)	0.284
Stroke history = Yes (%)	106 (19.8)	53 (17.6)	40 (22.2)	13 (23.6)	0.352
Clinical characteristics					
NIHSS (mean (SD))	3.51 (3.02)	2.55 (2.29)	4.46 (3.33)	5.58 (3.44)	<0.001
mRS (mean (SD))	2.09 (1.36)	1.68 (1.17)	2.42 (1.39)	3.27 (1.25)	<0.001
Barthel (mean (SD))	79.66 (26.25)	87.79 (20.62)	72.31 (28.15)	59.27 (29.45)	<0.001
SSRS (mean (SD))	37.08 (9.36)	36.18 (9.46)	38.08 (9.32)	38.80 (8.45)	0.035

PSD—post-stroke depression, BMI—body mass index, CHD—coronary heart disease, NIHSS—The National Institutes of Health Stroke Scale, mRS—modified rankin scale, BI—Barthel index, SSRS—social support rating scale.

### Transitions between post-stroke depressive states

Table 2 summarizes the frequency of transitions from the current state to the next state observed at four follow-up visits after stroke (baseline, 3 months, 6 months, and 12 months). Among them, 1,441 transitions of PSD state were observed, including 160 indicating progression forward, 244 indicating recovery backward, and 1,037 transitions remaining in the original state. For current mild-PSD patients, 40% recovered to non-PSD at the next follow-up, and 4.2% progressed to severe-PSD. For current severe-PSD patients, 26.3% recovered to non-PSD, and 42.2% recovered to mild-PSD. For current non-PSD patients, 14.6% progressed to possible mild-PSD, and 1.1% progressed to severe-PSD.

**Table 2:** Observed numbers of post-stroke depressive states transitions from one follow-up to the next follow-up

		Post-transition, n (%)		
		Non-PSD	Mild-PSD	Severe-PSD
Pre-transition, n (%)	non-PSD	756 (84.2)	131 (14.6)	10 (1.1)
	mild-PSD	176 (40.0)	250 (56.2)	19 (4.2)
	severe-PSD	26 (26.3)	42 (42.4)	31 (31.3)

The estimated transition intensities between different PSD states are presented in Table 3. The transition intensity for individuals with mild-PSD returning to non-PSD (0.165, 95% CI 0.138–0.196) was substantially higher than the intensity for worsening to severe-PSD (0.030, 95% CI 0.017–0.053). The transition intensity for non-PSD individuals worsening to mild-PSD (0.060, 95% CI 0.049–0.074) was higher than that for mild-PSD individuals worsening to severe-PSD (0.031, 95% CI 0.017–0.053).

**Table 3:** Transition intensity between different post-stroke depressive states

From\To	Non-PSD	Mild-PSD	Severe-PSD
Non-PSD	–	0.060 (0.049, 0.074)	0.003 (0.0008,0.012)
Mild PSD	0.165 (0.138,0.196)	–	0.030 (0.017,0.053)
Severe PSD	0.066 (0.024,0.181)	0.306 (0.211,0.444)	–

### Probability of post-stroke depressive state transitions

**Figure 1:**
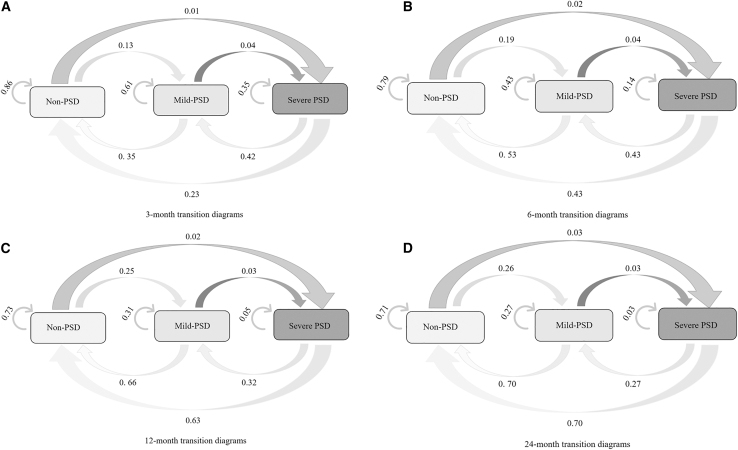
Transition diagrams of post-stroke depressive states within 3, 6, 12, and 24-month observation intervals The state transition diagrams for 3-month (A), 6-month (B), 12-month (C), and 24-month (D) observation intervals based on the MSM model, respectively.

Estimated probabilities of state transitions for individuals in different depressive states over observation intervals of 3, 6, 12, and 24 months (Figure 1). Specifically, for mild-PSD individuals, the estimated probabilities of recovering to non-PSD, remaining as mild-PSD, and worsening to severe-PSD were 0.66, 0.31, and 0.03, respectively, within 1 year of observation, and evolved to 0.70, 0.27, and 0.03, respectively, within 2 years of observation. For severe-PSD individuals, the estimated probabilities of recovering to non-PSD, recovering to mild-PSD, and remaining as severe-PSD were 0.63, 0.32, and 0.05, respectively, within 1 year of observation, and evolved to 0.70, 0.27, and 0.03, respectively, within 2 years of observation. For non-PSD, the estimated probabilities of remaining as non-PSD, worsening to mild-PSD, and worsening to severe-PSD were 0.73, 0.25, and 0.02, respectively, within 1 year of observation, and evolved to 0.71, 0.26, and 0.03, respectively, within 2 years of observation. The specific data on estimated state transition probabilities are provided in Figure 1.

### Covariate effects on post-stroke depressive state transitions

The covariates in the study include age, gender, BMI, marital status, education level, smoking, alcohol consumption, diabetes, hypertension, hyperlipidemia, coronary heart disease, history of stroke, mRS score, BI score, SSRS score, and NIHSS score. Univariate MSM models identified education, social support, drinking history, functional independence, and smoking as significantly associated with PSD state transitions (Table S3). Subsequently, a multivariate MSM model simultaneously evaluated covariates affecting both worsening and recovery transitions. Higher education (≥10 years) and stronger social support (SSRS >33) were independently associated with reduced risk of transitioning from non-PSD to mild-PSD (HRs 0.63, 95% CI 0.40–0.98; and 0.61, 95% CI 0.40–0.94). Drinking history was associated with increased likelihood of recovering from mild-PSD to non-PSD (HR 1.72, 95% CI 1.18–2.51), whereas functional dependence (BI score ≤60) predicted lower recovery probability (HR 0.68, 95% CI 0.46–0.99) (Table 4).

**Table 4:** Multivariate MSM model of post-stroke depressive state transitions

Characteristic	HR (95%CI)
Non-PSD to mild-PSD	
Education ≥10	0.63 (0.40–0.98)
SSRS >33	0.61 (0.40–0.94)
Mild-PSD to non-PSD	
Drinking	1.72 (1.18–2.51)
BI score ≤60	0.68 (0.46–0.99)

SSRS—social support rating scale, BI—Barthel index.

### Sojourn time of each transient state

**Figure 2:**
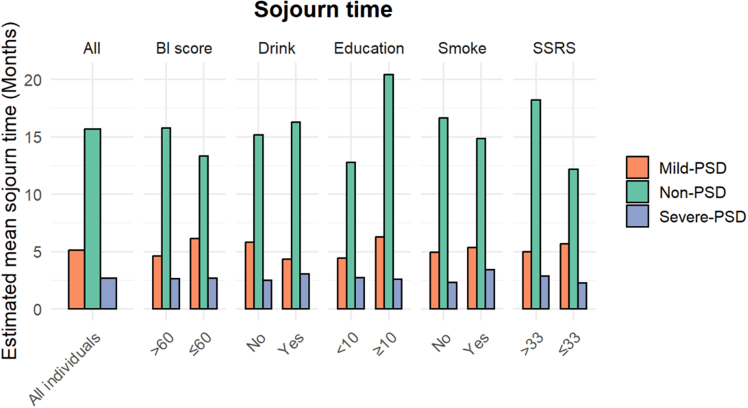
The estimated mean sojourn time of each PSD transient state before the next transition for the overall population and subgroups

The average duration of stay for each transient state was estimated in the overall population and subgroups. In the total population, the average duration of stay in the normal state was 15.70 months, in the mild-PSD state was 5.11 months, and in the severe-PSD state was 2.69 months (Figure 2). These sojourn time estimates assume no unobserved competing events between scheduled assessments. In the absence of death as an absorbing state, the mean sojourn time in severe-PSD may be underestimated if unobserved deaths occurred in this high-risk subgroup. The specific data on the estimated average duration of stay are provided as Table S2.

The observed and simulated changes in the prevalence of each state constructed in this study’s multistate Markov model are shown in Figure S3. Overall, our model captures the trend of changes in prevalence over time, which are similar.

### Sensitivity analysis

Sensitivity analyses using multiply-imputed datasets yielded consistent estimates for key predictors in both univariate and multivariate models (Tables S4–S8). Continuous variable specifications and adjustment for hospital site produced stable estimates (Table S5). The four-state model revealed overlapping sojourn time estimates between moderate and severe categories (Table S7).

## Discussion

This study, by constructing a multistate Markov model, provides an in-depth analysis of the fluctuation process of PSD states in Chinese stroke survivors and identifies key predictive factors influencing the transition of PSD states. Our findings emphasize that the PSD state of stroke survivors can transition bidirectionally, and a significant portion of mild and severe PSDs exhibit potential for recovery. Important factors, including years of education, drinking history, level of social support, and BI scores, influence the intensity of PSD state transitions. These findings provide a crucial theoretical basis for developing targeted early screening and intervention strategies, which can help reduce the long-term disease burden of PSD.

The results of this study are consistent with previous systematic review, revealing the dynamic natural course of PSD, characterized by the coexistence of new cases and episodes of depression remission.^14^ By constructing a multistate Markov model, we quantified the transition intensity and probability between different states, further elucidating the dynamic process of PSD.^15^ The study found that the developmental path of PSD exhibits diverse characteristics, manifesting as either gradual changes or abrupt transformations.^12^^,^^16^^,^^17^ It is worth noting that the transition strength between adjacent states is significantly higher than that between non-adjacent states, which may be closely related to the severity of symptoms and cumulative effects. Additionally, this study evaluated the potential transition probability of the PSD state over a relatively long period and found that a considerable proportion of mild and severe PSD patients have high recovery potential.^1^^,^^3^ Therefore, considering the diversity of depressive transition patterns, the multistate Markov model has higher accuracy in predicting the characteristics and progression paths of PSD courses, not only helping us understand their natural course but also providing important references for regular screening and the development of time-targeted intervention measures.

In the analysis of covariate effects, we found that an increase in education level significantly reduced the risk of worsening from a normal state to mild depression. This finding is consistent with previous studies, where education level is generally considered an important factor affecting the development of PSD symptoms.^18^^,^^19^ Patients with lower education levels may face a higher risk of depression due to cognitive biases, life stress, and limited social resources.^20^ Specifically, patients with lower educational levels often lack sufficient understanding of stroke and its consequences, fail to recognize symptoms correctly or intervene in a timely manner, leading to the emergence and development of negative emotions, as well as more severe health consequences.^21^ Furthermore, insufficient social support due to low education level (such as lack of economic and medical resources) exacerbates patients’ sense of isolation and stress, increasing the likelihood of depression.^6^^,^^22^ However, it should be noted that education level is not a static indicator and may not fully reflect a patient’s subsequent cognitive efforts. Therefore, during the treatment period, healthcare workers should actively provide stroke and stroke service-related information to stroke patients with lower education levels, thereby enhancing their ability to cope with the disease and reducing the occurrence of depressive symptoms.^23^^,^^24^

We observed an association between alcohol consumption and higher likelihood of recovery from mild-PSD (adjusted HR = 1.72, 95% CI 1.18–2.51) after controlling for baseline BI and social support. However, this observational finding should not be interpreted as causal. Residual confounding from unmeasured factors—such as premorbid functional status, cognitive reserve, or quality of social relationships—may explain this association. Alternatively, the finding may reflect social participation rather than alcohol per se, as drinking in Chinese culture predominantly occurs in social contexts.^25^^,^^26^ We explicitly caution against clinical recommendations for alcohol use. Even if the observed association were causal, the well-established harms of alcohol—including increased blood pressure, stroke recurrence risk^27^ and potential for dependence—outweigh any speculative benefits. Clinicians should instead focus on promoting non-alcoholic social activities and proven interventions such as physical exercise and psychosocial support.

Recent meta-analyses and empirical studies have shown that reduced social support is significantly associated with higher PSD symptoms.^28^^,^^29^ Consistent with previous randomized controlled trial studies, this study found that a higher level of social support helps promote the resolution of PSD.^30^ By meeting the social support components that patients value, including emotional support (a sense of being loved and accepted), tangible support (fostering independence), and the ability to participate, it can help patients rebuild their belief in their ability to overcome the disease, enhance social engagement and connection, thereby promoting functional recovery and reducing levels of psychological distress and unmet spiritual needs.^31^^,^^32^^,^^33^^,^^34^^,^^35^ However, the relationship between social support and PSD is difficult to explain. Excessive support may instead trigger patients’ feelings of self-blame or burden. For example, although family support is typically continuous and reliable for patients, it may be accompanied by underlying tension or disharmony.^31^ In China, parents’ love for their children is often based on giving and dedication, so stroke patients may feel a sense of guilt about becoming a burden to their children.^36^ Despite these challenges, strategies focused on improving comprehensive social support sources remain effective in managing emotional issues and supporting functional recovery.^30^^,^^35^^,^^37^ Healthcare professionals, stroke caregivers, and members of the community can improve patients’ support levels, mental health, quality of life, and satisfaction by increasing “behavioral activation” (engaging in valuable and beneficial activities) and “self-efficacy” (sense of competence and empowerment), thereby promoting recovery from the disease and reducing the socioeconomic burden.^38^^,^^39^

The severity of daily living activity impairment after stroke is one of the most closely related influencing factors to PSD.^14^^,^^40^ The BI is commonly used to assess the independence of ADL in stroke patients.^41^ The results of this study indicate that patients with daily living ability dependent (BI score ≤60) find it more difficult to recover from mild PSD to a normal state compared to independent patients with BI scores greater than 60. Patients with lower self-care ability often face both physical and psychological challenges, and psychological stress responses may lead to a gap between their actual perception and psychological experience, making them more prone to depressive symptoms.^19^^,^^42^^,^^43^ Furthermore, the impact of dependence on daily living activities on depression tends to increase over time, and symptoms may recur, especially when functional recovery reaches a plateau.^1^^,^^44^^,^^45^ Research comparing patients with persistent depression and non-persistent depression found that over time, the absolute degree of functional disability and poorer health status are associated with persistent and progressive depressive symptoms.^1^^,^^46^ Therefore, clinical healthcare professionals should encourage patients to actively participate in rehabilitation and implement comprehensive intervention measures based on the specific condition of the patient, especially for patients with moderate to severe disabilities, as improved functional activities can reduce the risk of PSD.

Our findings enable individualized risk stratification at admission that challenges the current uniform approach to PSD management. The distinct dynamic profiles of mild versus severe depression suggest divergent clinical strategies. For mild-PSD, the 5.5-fold higher transition intensity toward remission versus progression, combined with a 66% probability of spontaneous recovery within 12 months, supports an initial non-pharmacological strategy—prioritizing functional rehabilitation and structured monitoring over immediate antidepressant initiation. Conversely, severe-PSD exhibits a short mean sojourn time of 2.7 months with high exit rates. We caution that this apparent transience may be partially attributable to unobserved competing events, including mortality or dropout, which we could not model as absorbing states. If unobserved deaths occurred in this high-severity subgroup, the true sojourn time in severe-PSD may be longer and the recovery rate lower than estimated. Nevertheless, the high observed transition probabilities toward milder states and the absence of systematic baseline differences between completers and non-completers (Table S6) suggest that genuine recovery contributes substantially to these transitions. Pending validation with mortality-linked data, severe-PSD appears to represent a potentially transient but critical window that may warrant intensive, time-limited intervention. Quantitative thresholds (BI > 60, SSRS>33) identify patients likely to respond to short-course therapy, whereas those with functional dependence may require prolonged maintenance. Future prospective studies should validate these risk strata against clinical outcomes.

### Limitations of the study

This study has several limitations. First, the Markov assumption that transitions depend only on the current state may not fully capture history-dependent effects such as cumulative depression duration or multiple recurrences, particularly given the 12-month follow-up period. Second, we did not have mortality data and could not explicitly model death or loss to follow-up as absorbing or competing states in the multistate Markov model. This omission is methodologically consequential: if patients in severe-PSD died or dropped out at higher rates than those in milder states, the estimated transition intensities from severe-PSD to mild-PSD or non-PSD would be artificially inflated, and the average sojourn time in severe-PSD (2.69 months) would be underestimated. In other words, the apparent transience of severe-PSD may partly reflect unobserved attrition rather than genuine rapid remission. Although baseline comparability between completers and non-completers provides some reassurance, we cannot exclude informative dropout or mortality selection. Consequently, recovery estimates from severe-PSD should be regarded as hypothesis generating, and clinical conclusions about the state’s transience require validation in datasets that explicitly capture mortality and long-term attrition. Third, as an observational study, we cannot establish causality between predictors and depression transitions, and residual confounding from unmeasured factors may persist despite multivariate adjustment. Fourth, we did not formally test for interactions between covariates or non-linear relationships beyond clinically selected cut-points. Fifth, dichotomization of continuous variables involves inherent trade-offs between statistical efficiency and clinical interpretability; although sensitivity analyses using continuous specifications confirmed robustness, we did not model spline terms or threshold effects. Sixth, detailed lesion location data were not systematically recorded, precluding analysis of whether anatomical factors modify functional recovery effects on remission. Seventh, the lack of continuous monitoring between scheduled visits hinders precise estimation of transition times. Eighth, constrained by depression assessment methods, we excluded patients with aphasia or severe cognitive impairment, potentially resulting in a cohort with milder stroke severity than the general population and affecting generalizability to severe cases. Ninth, the 12-month follow-up may not fully capture the chronic course of PSD, including late-onset depression or recurrence beyond one year.

## Resource availability

### Lead contact

Requests for further information and resources should be directed to and will be fulfilled by the lead contact, Zhou Zhu (zhouzhu@hust.edu.cn).

### Materials availability

This study did not generate new unique reagents.

### Data and code availability

• Data reported in this paper will be shared by the lead contact upon request.
• This paper does not report original code.
• Any additional information required to reanalyze the data reported in this paper is available from the lead contact upon request.

## Acknowledgments

We would like to thank all participants who voluntarily participated in this study. Funding: 10.13039/501100001809National Natural Science Foundation of China (grant no 82171465) and Wuhan Key Research and Development Program Project (grant no 2025020102030017).

## Author contributions

Conceptualization, data curation, methodology, formal analysis, writing – original draft, writing – review and editing, W.L.; data curation, methodology, formal analysis, writing – review and editing, Y.F.; supervision, conceptualization, T.L.; methodology, Q.D.; visualization, Y.L.; validation, S.C.; conceptualization, project administration, C.P.; conceptualization, supervision, project administration, G.L.; conceptualization, funding acquisition, supervision, project administration, Z.Z. All authors have read and approved the final version of the manuscript. The corresponding authors attest that all listed authors meet authorship criteria and that no others meeting the criteria have been omitted.

## Declaration of interests

The authors declare no competing interests.

## STAR★Methods

### Key resources table

**Table:** 

REAGENT or RESOURCE	SOURCE	IDENTIFIER
**Software and algorithms**		

R studio version 4.5.1	R softwore	https://www.rstudio.com/tags/rstudio/
msm (R package)	Christopher Jackson et al.(2024)	https://github.com/chjackson/msm

### Experimental model and study participant details

#### Human participants

This multicenter cohort study was conducted at three hospitals in Wuhan, Hubei Province, China (Tongji Hospital, Wuhan First Hospital, and Wuhan Central Hospital).

Inclusion criteria were: (1) acute ischemic stroke confirmed by magnetic resonance imaging or computed tomography within 7 days of onset; (2) age ≥18 years. Exclusion criteria were: (1) non-vascular brain dysfunction (e.g., brain tumors, traumatic brain injury); (2) history of emotional disorders, dementia, or other psychiatric diseases; (3) communication disorders (aphasia, severe speech difficulties, comprehension impairment, or altered consciousness) precluding standardized assessment; (4) inability to complete follow-up assessments; (5) transient ischemic attack or subarachnoid hemorrhage; (6) concurrent neurological diseases including Parkinson’s disease or epilepsy.

Of 937 consecutively enrolled patients with ischemic stroke, 536 participants with at least two depression status assessments and complete baseline predictors were included in the analysis (Figure S1). The main reasons for participant loss were refusal to continue or loss to contact. This was an observational cohort study; participants were not allocated to experimental intervention groups.

#### Participant characteristics

Age, sex/gender, education, vascular risk factors, and stroke severity were recorded at baseline. Detailed demographic and clinical characteristics of the analyzed cohort are provided in Table 1. The cohort comprised male and female participants of East Asian ancestry (Chinese Han ethnicity); mean age was approximately 59 years. Race and ethnicity were not systematically cataloged using international standardized categories because the study was conducted in a single-country setting with a relatively homogeneous population. Socioeconomic status was not formally measured; years of education was used as a proxy indicator. Sex was included as a covariate in all Markov models to control for demographic confounding. However, we did not perform sex-stratified analyses or formally test for sex-by-covariate interactions on transition intensities. Therefore, whether the predictors of depression state transitions differ between males and females remains undetermined, which may limit the generalizability of our findings to specific sex or gender groups.

#### Ethics statement

All procedures were approved by the Ethics Committee of Tongji Medical College, Huazhong University of Science and Technology (approval number: TJ-IRB20171108) and comply with the Declaration of Helsinki. Written informed consent was obtained from all participants.

### Method details

#### Post-stroke depressive states

At baseline and during follow-ups at three, six and twelve months post-stroke, depression symptoms were measured using the 17-item Hamilton Depression Rating Scale (HRSD). The Chinese version of HRSD demonstrated high reliability and validity, serving as an indicator for assessing the severity of depression symptoms.^47^ HRSD consists of 17 items, with a score range of 0–54, where a higher score indicates more severe depressive symptoms. The HRSD assessment was conducted by two experienced psychiatrists after receiving standardized training. The inter-rater reliability reached an acceptable level. Our main goal is to study the transitions between various post-stroke depressive states (PSDS), so we categorized HRSD scores of 0–7 as No Post-Stroke Depression (Non-PSD), 8–16 as Mild Post-Stroke Depression (Mild-PSD), and scores above 17 as Severe Post-Stroke Depression (Severe-PSD, encompassing moderate-to-severe depression).^48^^,^^49^

#### Possible predictors

In our study, we considered three major categories of variables. Socio-demographic parameters, including age (years), gender (male/female), BMI (kg/m^2^), marital status (married/single), and years of education. Age was categorized into two groups: ≤65 years,>65 years. BMI was classified into three categories based on Chinese adult body weight: underweight, defined as BMI <18.5 kg/m^2^; normal weight, defined as 18.5 kg/m^2^ ≤ BMI <24.0 kg/m^2^; and overweight and obesity, defined as BMI 24.0 kg/m^2^.^50^ Education level is divided into 2 categories: <10 years, ≥10 years. Vascular risk factors, including smoking history (yes/no), alcohol history (yes/no), diabetes history (yes/no), hypertension history (yes/no), hyperlipidemia history (yes/no), coronary heart disease history (yes/no), and stroke history (yes/no). Clinical characteristics, including baseline mRS score, baseline BI score, baseline SSRS, and baseline NIHSS. Modified Rankin Scale (mRS): Patients are divided into 2 groups based on baseline mRS results: good (mRS 0–3) and poor (mRS 3–6).^51^ Barthel index (BI): Good (BI > 60 points), Poor (BI ≤ 60 points).^41^ Social Support Rating Scale (SSRS): The total score of 10 items ranges from 12 to 64, indicating two different levels of social support (12–33 points indicate a low level, 34–64 points indicate a high level).^52^ National Institutes of Health Stroke Scale (NIHSS): Mild stroke ≤5 points; Moderate to severe stroke >5 points.^53^ Missing data are described in Table S1. Multiple imputation was used to fill missing values for subsequent analysis. All predictors were baseline measurements obtained at hospital admission, consistent with our prognostic objective of early risk stratification for subsequent depression state transitions.

### Quantification and statistical analysis

Descriptive analysis is used to describe sociodemographic and clinical data. For continuous data, mean and standard deviation are used; for categorical data, frequency and percentage are used. Based on the Kolmogorov-Smirnov test for normal or nonnormal distribution of data, the *t* test or Mann-Whitney U test is used for comparison. Categorical variables are presented as frequency and percentage and analyzed using the chi-square test.

A multistate model is a continuous-time stochastic process model in which participants may experience a limited number of clinical states during follow-up. This model can comprehensively represent the disease progression and is helpful for potential factor analysis and long-term prediction.^54^ Fitting a multistate Markov model to panel data typically relies on the Markov assumption, that future evolution depends only on the current state and not on any previous transitions.^9^ We assumed a first-order Markov process where transition probabilities depend only on the current depression state. This assumption is supported by our exclusion of patients with prior emotional disorders (ensuring first-onset PSD) and the 12-month follow-up duration, though we acknowledge it may not capture long-term chronicity effects.Given the dynamic nature of PSD and referring to previous studies,^12^^,^^55^ we constructed a hypothetical state transition diagram for PSD disease based on a Markov process. As shown in Figure S2, we defined three possible states (non-PSD marked as state 1, mild-PSD marked as state 2, and severe-PSD marked as state 3) and assumed that individuals can progress or recover between adjacent disease states. Based on this three-state model, the corresponding transition intensity matrix Q is represented as follows: $Q=\left(\begin{array}{ccc} -\left(q_{12}+q_{13}\right) & q_{12} & q_{13}\\ q_{21} & -\left(q_{21}+q_{23}\right) & q_{23}\\ q_{31} & q_{32} & -\left(q_{31}+q_{32}\right) \end{array}\right)$

Here, the subsequent states to which participants transition are determined by the transition intensity matrix Q. Although panel data is considered as process snapshots, the intensity symbolizes the instantaneous risk of transitioning from one state to another.^9^ By introducing regression components into the intensity matrix Q, other variables related to the topic, such as demographic characteristics and clinical functional assessments, can be combined. Transition probabilities describe the probability of a certain transition occurring between possible states at a given time, calculated using the matrix exponential of the transformation intensity matrix Q. Average dwell time refers to the average time spent in a transient state before transitioning to the next state. The diagonal elements of the estimated transition intensity matrix can be predicted using the relationship −1/qrr to determine the average dwell time for each transient state.

In this study, multistate Markov analysis was conducted in three steps. First, a Markov model without covariates was performed to estimate the transition intensity and probabilities between states, as well as the average residence time in each state. Second, a univariate MSM model was used to test the effect of covariates on state transitions, estimating the hazard ratio of covariates on transition intensity and its 95% confidence interval. Third, a multivariate MSM model was established, including covariates that significantly affected depressive state transitions. Model fit was assessed by comparing observed state prevalences at each follow-up visit with model-predicted prevalences derived from 1000 Monte Carlo simulations (Figure S3). All analyses used two-sided tests, with statistical significance set at *p* < 0.05. Data analyses were performed using R software (version 4.5.1).

#### Sensitivity analysis

1. We use multiply imputed datasets from the mice package to assess the robustness of covariate effects. 2. To assess whether binary categorization masked underlying linear relationships or threshold effects, we conducted sensitivity analyses treating BI and SSRS as standardized continuous variables (z-scores) in the multivariate Markov model. These analyses aimed to validate the robustness of findings from binary specifications. 3. To evaluate potential inter-site variability, we conducted a sensitivity analysis including hospital site (Tongji Hospital, Wuhan First Hospital, Wuhan Central Hospital) as a categorical covariate in the multivariate Markov model. This assessed whether differences in clinical management protocols between the three centers influenced the estimated transition intensities.4.While the primary analysis collapsed moderate (HRSD 17–23) and severe (≥24) depression into a single “Severe-PSD” state due to limited sample size in the ≥24 group, we additionally fitted a four-state Markov model (non-PSD, mild-PSD, moderate-PSD, severe-PSD) as a sensitivity analysis. This evaluated whether the two severe categories exhibited distinct transition dynamics or sojourn times that would challenge their consolidation.
