# Supplementary material for: Predictive factors for remission in post-stroke depression: A Markov model cohort study
Source: iScience. 2026 Jun 17;29(7):116438. doi: 10.1016/j.isci.2026.116438 (PMC13293725; doi:10.1016/j.isci.2026.116438)
Supplement: Document S1. Figures S1–S3 and Tables S1–S8 [file mmc1.pdf]

iScience, Volume *29*

Supplemental information

Predictive factors for remission in post-stroke depression: A Markov model cohort study

Wenwen Liang, Yifan Fang, Tianyi Li, Qing Du, Yingjie Liu, Siyi Chen, Chensheng Pan, Guo Li, and Zhou Zhu

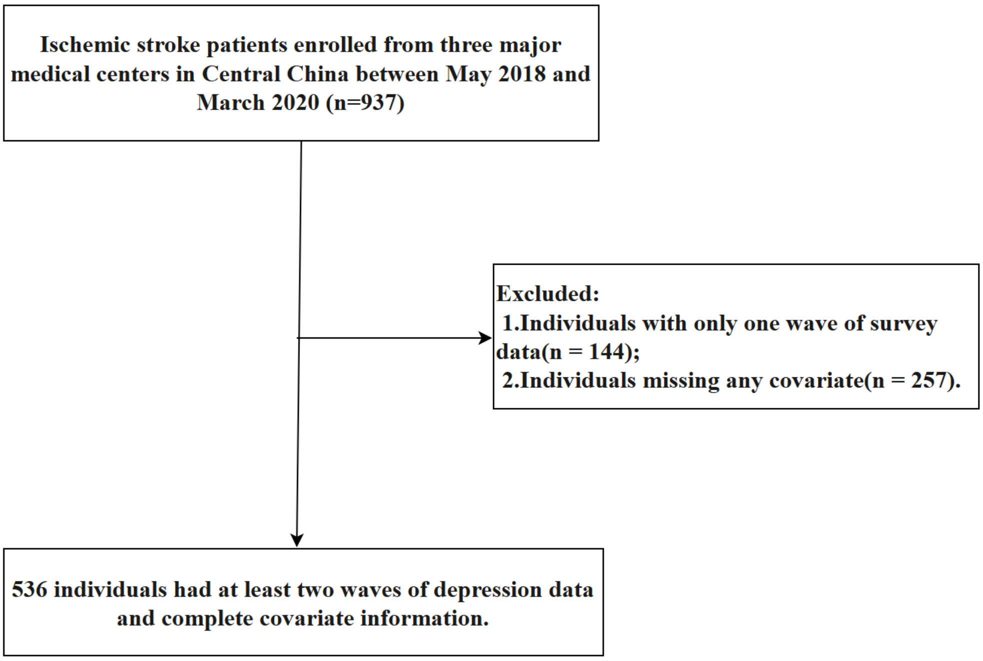

Supplementary Figure 1. Cohort sample selection flowchart.

Related to STAR Methods.

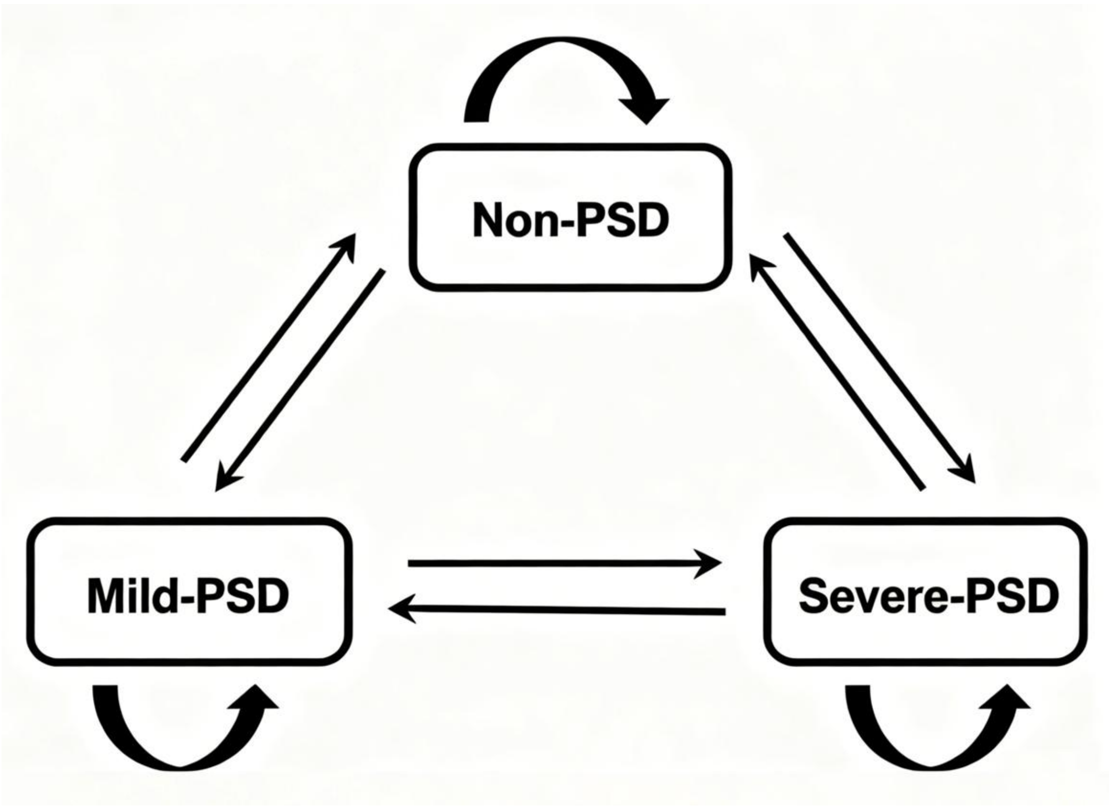

Supplementary Figure 2. Hypothesized diagram showing the PSD state transition,

based on the Markov process.

Legend: PSD =Post-stroke depression.

Related to Figure 1.

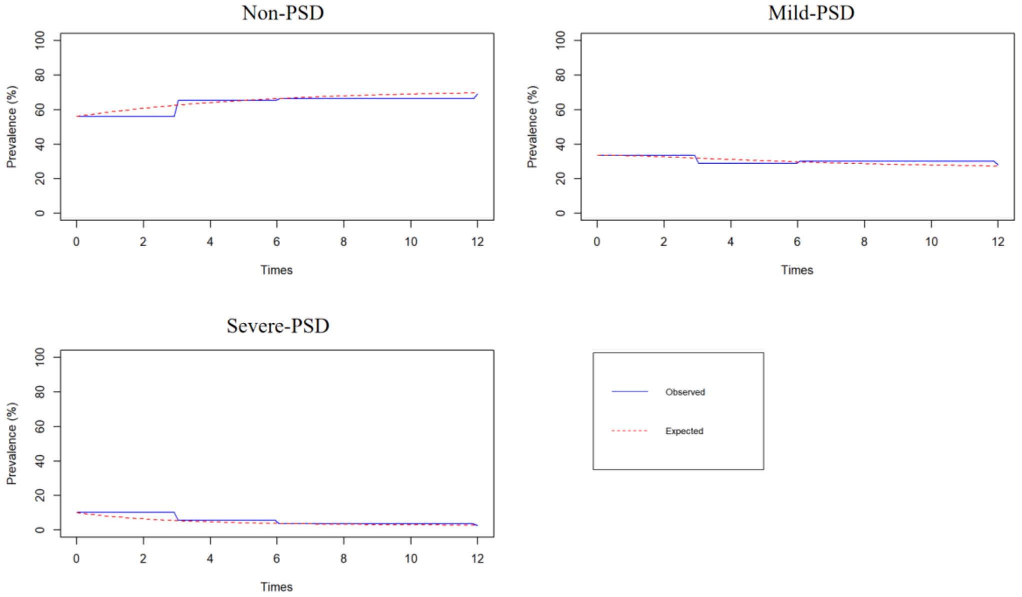

Supplementary Figure 3. Observed (solid line) and simulated (dotted line) prevalence transition of each state.

Legend: The figure demonstrates the observed and simulated prevalence transition of each state from three multi-state Markov models constructed in this study.

Related to STAR Methods.

Supplementary Table 1. The distribution of missing values among all records of enrolled individuals.

Related to STAR Methods.

Distribution s of missing values, n (%)	Total records = 793
Sex	1 (0.13)
Age	8 (1.01)
BMI	58 (7.31)
Education	44(5.55)
Married	26 (3.28)

Smoking	16 (2.02)
Drinking	18 (2.27)
Diabetes mellitus	48 (6.05)
Hypertension	48 (6.05)
Dyslipidemia	118 (14.9)
CHD	59 (7.44)
Stroke history	48 (6.05)
NIHSS	2 (0.25)
mRS	10 (1.26)
Barthel index	13 (1.64)
SSRS	27 (3.4)

Legend: BMI Body mass index, CHD Coronary heart disease, NIHSS The National Institutes of Health Stroke Scale, mRS Modified Rankin Scale, BI Barthel index, SSRS Social Support Rating Scale.

Supplementary Table 2. The estimated mean sojourn time before the next transition and predicted total stay of each post-stroke depression transient state among different subgroups

Related to Figure 2.

Variable	Group	Depressive States	Sojourn time(months)
All	All individuals	Non-PSD	15.7
All	All individuals	Mild-PSD	5.11
All	All individuals	Severe-PSD	2.69
Education	≥10	Non-PSD	20.45
Education	≥10	Mild-PSD	6.27
Education	≥10	Severe-PSD	2.57
Education	<10	Non-PSD	12.79
Education	<10	Mild-PSD	4.41
Education	<10	Severe-PSD	2.72
SSRS score	>33	Non-PSD	18.20
SSRS score	>33	Mild-PSD	4.97
SSRS score	>33	Severe-PSD	2.86

SSRS score	≤33	Non-PSD	12.19
SSRS score	≤33	Mild-PSD	5.68
SSRS score	≤33	Severe-PSD	2.25
Smoking	Yes	Non-PSD	14.84
Smoking	Yes	Mild-PSD	5.35
Smoking	Yes	Severe-PSD	3.42
Smoking	No	Non-PSD	16.63
Smoking	No	Mild-PSD	4.92
Smoking	No	Severe-PSD	2.29
Drinking	Yes	Non-PSD	16.27
Drinking	Yes	Mild-PSD	4.35
Drinking	Yes	Severe-PSD	3.07
Drinking	No	Non-PSD	15.16
Drinking	No	Mild-PSD	5.8
Drinking	No	Severe-PSD	2.48
BI score	>60	Non-PSD	15.77
BI score	>60	Mild-PSD	4.61
BI score	>60	Severe-PSD	2.63
BI score	≤60	Non-PSD	13.34
BI score	≤60	Mild-PSD	6.16
BI score	≤60	Severe-PSD	2.67

Legend: SSRS Social Support Rating Scale, BI Barthel index.

Supplementary Table 3. Univariate MSM model of covariate effects on post-stroke depressive state transitions.

Related to Results.

Transition	Transition	HR (95% CI)
Non-PSD to Mild-PSD	Education ⩾10 years	0.61 (0.40–0.92)
Non-PSD to Mild-PSD	SSRS >33	0.60 (0.40–0.90)
Mild-PSD to Non-PSD	Drinking	1.48 (1.04–2.10)
Mild-PSD to Non-PSD	BI score ⩽60	0.64 (0.43–0.95)
Severe-PSD to Mild-PSD	Smoking	0.36 (0.15–0.87)

Legend: HR, hazard ratio; CI, confidence interval; SSRS, Social Support Rating Scale; BI, Barthel index.

Supplementary Table 4. Baseline characteristics of the participants (covariates with interpolated values).

Related to Sensitivity Analysis (Results).

Variables	Total (n=793)	Non-PSD (n=412)	Mild-PSD (n=270)	Severe-PSD (n=109)	P
Sociodemographic parameters					
Sex = Male (%)	616 (77.7)	328(79.6)	215(79.6)	70(64.2)	0.002
Age (mean (SD))	58.62 (11.03)	59.41(11.17)	57.66(11.37)	57.94(9.67)	0.105
BMI (mean (SD))	24.78 (3.70)	24.79(3.99)	24.77(3.93)	24.35(3.25)	0.551
Education (%)≥10 years	323（40.7）	183(44.4)	100(37.0)	38(34.9)	0.067
Married (%)	782 (98.6)	406(98.5)	267(98.9)	108 (99.1)	0.870
Vascular risk factors					
Smoking = Yes (%)	357 (45.0)	195 (47.3)	117 (43.3)	41 (37.6)	0.168
Drinking = Yes (%)	345 (43.5)	168 (40.8)	129 (47.8)	45 (41.3)	0.178
Diabetes mellitus = Yes (%)	223 (28.1)	127 (30.8)	72 (26.7)	26 (23.9)	0.260
Hypertension= Yes (%)	478 (60.3)	258 (62.6)	164 (60.7)	69 (63.3)	0.850
Dyslipidemia = Yes (%)	211 (26.6)	112 (27.2)	73 (27.0)	37(33.9)	0.339
CHD = Yes (%)	82 (10.3)	35 (8.5)	28 (10.4)	18 (16.5)	0.049
Stroke history = Yes (%)	169 (21.3)	84 (20.4)	60 (22.2)	26 (23.9)	0.690
Clinical characteristics					
NIHSS (mean (SD))	3.71 (3.26)	2.64 (2.57)	4.37 (3.26)	6.06 (3.86)	<0.001
mRS (mean (SD))	2.20 (1.37)	1.72(1.17)	2.46 (1.36)	3.34 (1.25)	<0.001
Barthel (mean (SD))	78.46 (27.01)	87.86 (21.18)	72.89	56.74	<0.001

			(27.51)	(28.95)	
SSRS (mean (SD))	37.74 (8.93)	36.92 (9.21)	38.47 (8.75)	38.62 (8.01)	0.043

Legend: PSD Post-stroke depression, BMI Body mass index, CHD Coronary heart disease, NIHSS The National Institutes of Health Stroke Scale, mRS Modified Rankin Scale, BI Barthel index, SSRS Social Support Rating Scale.

Supplementary Table 5. Multivariate MSM model sensitivity analyses: multiple imputation, continuous variable specifications, and hospital site adjustment.Related to Sensitivity Analysis (Results).

Transition	Characteristic	Main model (binary)	Multiple imputation	Continuous variable	Adjusted for hospital site
Non-PSD to Mild-PSD	Education ≥10 years	0.63 (0.40–0.98)	0.67 (0.46–0.99)	—	0.68 (0.47–0.99)
Non-PSD to Mild-PSD	SSRS	—	—	0.69 (0.54–0.89)	—
Mild-PSD to Non-PSD	Drinking	1.72 (1.18–2.51)	1.56 (1.16–2.09)	—	1.78 (1.26–2.53)
Mild-PSD to Non-PSD	BI score	—	—	1.20 (1.01–1.42)	—
Mild-PSD to Non-PSD	BI score ≤60	0.68 (0.46–0.99)	0.67 (0.49–0.91)	—	0.70 (0.48–1.00)
Mild-PSD	SSRS >33	—	—	—	1.66 (1.08–

to Non-PSD					2.57)

Legend: HR, hazard ratio; CI, confidence interval; SSRS, Social Support Rating Scale; BI, Barthel index. The main model (binary) uses the same specifications as the primary analysis in Table 3. The continuous variable column shows HRs for standardized continuous predictors. The hospital site adjustment column includes Tongji Hospital, Wuhan First Hospital, and Wuhan Central Hospital as a categorical covariate.

Supplementary Table 6. Baseline characteristics of completers and non-completers. Related to STAR Methods / Limitations.

Variables	Completer (n=536)	Non-Completer (n=401)	P
Sex = Male (%)	423(78.9)	296(74.0)	0.092
Age (mean (SD))	59.12(11.08)	57.75(10.75)	0.068
BMI (mean (SD))	24.80(3.91)	24.63(3.25)	0.777
Education (%)⩾10 years	228(42.5)	110(35.0)	0.037
Married (%)	526(98.1)	347(98.9)	0.567
Smoking = Yes (%)	244 (45.5)	155 (46.1)	0.916
Drinking = Yes (%)	227 (42.4)	150 (44.9)	0.502
Diabetes mellitus = Yes (%)	152 (28.4)	80 (26.5)	0.617
Hypertension= Yes (%)	316 (59.0)	210 (69.5)	0.003
Dyslipidemia = Yes (%)	131 (24.5)	72 (31.7)	0.048
CHD = Yes (%)	50 (9.3)	30 (10.5)	0.687

Stroke history = Yes (%)	106 (19.8)	65 (21.5)	0.608
NIHSS (mean (SD))	3.5 (3.02)	4.04 (3.66)	0.103
mRS (mean (SD))	2.09(1.36)	2.32 (1.35)	0.005
Barthel (mean (SD))	79.69 (26.25)	77.04 (27.56)	0.144
SSRS (mean (SD))	37.07 (9.34)	38.00 (8.46)	0.158

Legend: BMI Body mass index, CHD Coronary heart disease, NIHSS The National Institutes of Health Stroke Scale, mRS Modified Rankin Scale, BI Barthel index, SSRS Social Support Rating Scale.

Supplementary Table 7. Four-state Markov model sensitivity analysis comparing moderate (17–23) and severe (⩾24) depression states.

Related to Sensitivity Analysis (Results).

	Moderate-PSD	Severe-PSD
Baseline n (%)	44(8%)	11(2%)
Mean sojourn time, months (95%CI)	1.58 (1.10–2.25)	1.55 (0.75–3.17)
Transition intensities		
To Non-PSD	0.082 (0.02–0.255)	0.0009 (8.1×10^{-11}–1.1×10^{4})
To Mild-PSD	0.427 (0.28–0.652)	0.003 (4.1×10^{-10}–2.3×10^{4})
To adjacent severe (Severe-PSD→Moderate-PSD or Moderate-PSD→Severe-PSD)	0.125 (0.04–0.367)	0.643 (0.311–1.327)

Supplementary Table 8. Univariate MSM models with multiply-imputed datasets. Related to Sensitivity Analysis (Results).

Transition	Transition	HR (95% CI)
Non-PSD to Mild-PSD	Education ⩾10 years	0.64 (0.45–0.90)
Non-PSD to Mild-PSD	SSRS >33	0.73 (0.52–1.00)
Mild-PSD to Non-PSD	Drinking	1.44 (1.12–1.87)
Mild-PSD to Non-PSD	BI score ⩽60	0.63 (0.45–0.88)
Severe-PSD to Mild-PSD	Smoking	0.47 (0.26–0.83)

Legend: HR, hazard ratio; CI, confidence interval; SSRS, Social Support Rating Scale; BI, Barthel index.
